# A pilot study of quality of life, mood, sleepiness and fatigue in patients with primary humoral immunodeficiency transitioning to subcutaneous immunoglobulin therapy

**DOI:** 10.1186/1710-1492-10-S2-A37

**Published:** 2014-12-18

**Authors:** Persia Pourshahnazari, Gina Tsai, Adriana Martin, Amin Kanani, Donald Stark, R Robert Schellenberg

**Affiliations:** 1 Department of Internal Medicine, University of British Columbia, Vancouver, British Columbia, Canada; 2 Department of Medicine, Division of Allergy & Immunology, Schulich School of Medicine & Dentistry, Western University, London, Ontario, Canada; 3 Department of Medicine, Division of Allergy and Clinical Immunology, University of British Columbia, Vancouver, British Columbia, Canada

## Background

Immunoglobulin replacement therapy is standard of care for patients with primary humoral immunodeficiency [1]. Compared with intravenous immunoglobulin (IVIG), subcutaneous immunoglobulin (SCIG) offers comparable efficacy, lower costs and reduced systemic reactions [2,3]. However, little is known about effects on quality of life when patients transition from IVIG to SCIG. It was our objective to assess changes in quality of life, mood, sleepiness and fatigue in patients transitioning from IVIG to SCIG.

## Methods

Adult patients with common variable immunodeficiency or X-linked agammaglobulinemia transitioning from IVIG to SCIG were invited to participate in this prospective, open-label, pilot study. At least one set of Short-Form 36 Health Survey (SF-36), Profile of Mood States (POMS), Epworth Sleepiness Scale (ESS) and nighttime sleep questionnaires was administered prior to the final IVIG infusion. These were repeated monthly for 3 months following the transition. Magnitude of change was estimated between IVIG trough and final SCIG steady-state data. Statistical significance was determined using linear mixed models for repeated measures with Kenward-Rogers correction.

## Results

Twenty-seven patients were included in the analysis. Two of eight SF-36 quality of life domains showed significant improvement: role limitations due to physical health (p = 0.01) and emotional problems (p = 0.04). Two of six POMS mood subscales significantly improved: depression (p = 0.03) and anger (p = 0.04). One of six POMS mood subscales (tension, p = 0.08) and POMS total mood disturbance scores (p = 0.09) trended towards improvement. No significant changes were noted in ESS or nighttime sleepiness scores.

## Conclusions

Patients transitioning from IVIG to SCIG for treatment of primary antibody immunodeficiency showed significant improvement in several quality of life and mood subscales. A larger study verifying these findings could encourage patients to switch to SCIG self-administration, producing quality of life benefits while decreasing health care costs.
